# Uterine Asthma

**Published:** 1856-05

**Authors:** 


*Uterine Asthma.*—Dr. Simpson has reported several cases of uterine asthma connected with menstruation. He made a few preliminary remarks on the observations of some German physiologists, with regard to the quantity of carbon excreted from the lungs during menstruation. Dr. Simpson had seen several cases of asthma, where the spasmodic attacks recurred at each menstrual period. In some, the difficulty of breathing was very great indeed; some, so much so, that, in one case in particular, the breathing could be heard from any part of the house; in other cases, where the catamenia were scanty, but present, the attacks were not so severe, the dyspnœa slight, but still recurring at the periods. Dr. S. mentioned the particulars of several cases, and stated that the dyspnœa was completely relieved by chloroform. Dr. S. wished the other members to assist him in investigating this subject.—*Edinburgh Medical Journal.*

A similar case was reported in this Journal, by Dr. Hoyt, which was cured by the iodide of potassium. See vol, liii, page 326.—*Boston Med. & Surg. Journal.*

